# D-Alanylation of Lipoteichoic Acids Confers Resistance to Cationic Peptides in Group B *Streptococcus* by Increasing the Cell Wall Density

**DOI:** 10.1371/journal.ppat.1002891

**Published:** 2012-09-06

**Authors:** Ron Saar-Dover, Arkadi Bitler, Ravit Nezer, Liraz Shmuel-Galia, Arnaud Firon, Eyal Shimoni, Patrick Trieu-Cuot, Yechiel Shai

**Affiliations:** 1 Department of Biological Chemistry, The Weizmann Institute of Science, Rehovot, Israel; 2 Department of Chemical Research Support, The Weizmann Institute of Science, Rehovot, Israel; 3 Institut Pasteur, Unité de Biologie des Bactéries Pathogènes à Gram-Positif, CNRS-ERL3526, Paris, France; 4 Electron Microscopy Unit, The Weizmann Institute of Science, Rehovot, Israel; University of Tubingen, Germany

## Abstract

Cationic antimicrobial peptides (CAMPs) serve as the first line of defense of the innate immune system against invading microbial pathogens. Gram-positive bacteria can resist CAMPs by modifying their anionic teichoic acids (TAs) with D-alanine, but the exact mechanism of resistance is not fully understood. Here, we utilized various functional and biophysical approaches to investigate the interactions of the human pathogen Group B *Streptococcus* (GBS) with a series of CAMPs having different properties. The data reveal that: (i) D-alanylation of lipoteichoic acids (LTAs) enhance GBS resistance only to a subset of CAMPs and there is a direct correlation between resistance and CAMPs length and charge density; (ii) resistance due to reduced anionic charge of LTAs is not attributed to decreased amounts of bound peptides to the bacteria; and (iii) D-alanylation most probably alters the conformation of LTAs which results in increasing the cell wall density, as seen by Transmission Electron Microscopy, and reduces the penetration of CAMPs through the cell wall. Furthermore, Atomic Force Microscopy reveals increased surface rigidity of the cell wall of the wild-type GBS strain to more than 20-fold that of the *dltA* mutant. We propose that D-alanylation of LTAs confers protection against linear CAMPs mainly by decreasing the flexibility and permeability of the cell wall, rather than by reducing the electrostatic interactions of the peptide with the cell surface. Overall, our findings uncover an important protective role of the cell wall against CAMPs and extend our understanding of mechanisms of bacterial resistance.

## Author Summary

Cationic antimicrobial peptides (CAMPs) represent important evolutionarily conserved elements of innate immunity and their killing mechanism involves bacterial cell wall permeation. As a result, gram-positive bacteria can resist CAMPs by modifying their anionic teichoic acids (TAs) following incorporation of D-alanyl residues to neutralize their surface charge, a reaction catalyzed by the *dlt* operon gene product. Here, we demonstrate that this electrochemical modification changes the barrier properties of Group B *Streptococcus* cell wall and inactivation of the *dlt* operon activity results in CAMP sensitivity. However, despite the major increase in the surface charge of the mutant, no increased electrostatic binding of CAMPs is observed. Rather, D-alanine incorporation protects the bacterial membrane by reducing the penetration of CAMPs through the cell wall. Accordingly, a *dlt* mutant was more susceptible to perforation by CAMPs and its cell wall nanostructure was significantly altered. Overall, we demonstrate a novel protective role of the cell wall against CAMPs which should enable bacterial invaders to survive upon host's colonization.

## Introduction

The innate immune system of almost all living organisms produce cationic antimicrobial peptides (CAMPs) to protect against bacterial invaders. CAMPs are gene-encoded peptides that differ in their primary amino acid sequences [1], [2] but most native CAMPs share a well-defined α-helix or β-strand secondary structures and display a net positive charge of mainly +2 to +9 [3], [4]. In contrast to conventional antibiotics that interact with specific targets, many CAMPs bind and perturb the bacterial membrane. Nevertheless, throughout evolution certain gram-positive bacteria have evolved sophisticated regulatory mechanisms to modify their surface properties in order to overcome killing by CAMPs. These are typified by two-component systems (TCSs) that sense and respond to environmental CAMPs [5]–[8].

The cell wall of gram-positive bacteria is a complex network composed mainly of peptidoglycan (PGN) and teichoic acids (TAs), both of which are essential for maintaining the structural integrity and shape of the bacterial cell. Teichoic acids are negatively charged poly-glycerophosphate (Gro-P) chains that can be either covalently linked to PGN (i.e. wall teichoic acids or WTAs) or anchored to the cytoplasmic membrane (i.e. lipoteichoic acids or LTAs) [9]. WTAs and LTAs are assembled *via* different pathways. The anionic property of teichoic acids confers a global negative charge, which is thought to contribute to the preferential accumulation of CAMPs on the bacterial cell surface [10]. Subsequently, these peptides traverse the PGN barrier, reach the anionic phospholipid of the cytoplasmic membrane, and perturb it *via* several mechanisms, depending on the peptide used [11]–[13].

In gram-positive bacteria, resistance to CAMPs is mainly due to an increase of the positive surface charge through increase in D-alanylation of teichoic acids (TAs) mediated by the *dlt* operon gene products and/or incorporation of L-lysine into phosphatidylglycerol, the major membrane lipid, mediated by the *mprF* gene product [10]. Deletion of the *dlt* operon leads to the complete absence of D-alanyl esters of TAs in *Staphylococcus aureus* (*S. aureus*) [14], Group A *Streptococcus* [15], *Streptococcus pneumoniae* [16], *Enterococcus faecalis* [17], and Group B *Streptococcus* (GBS) [14], which results in an increased susceptibility to various cationic antimicrobial agents. It has been demonstrated that lowering the anionicity of the cell wall causes a reduction in the electrostatic attraction between *S. aureus* and the cationic antibiotics gallidermin [14] and vancomycin [18]. Therefore, reduced electrostatic attraction represents one of the mechanisms by which D-alanylation of TAs can mediate CAMP resistance. However, the molecular mechanism by which D-alanylation of TAs mediates resistance has not been fully described.

GBS, also known as Streptococcus agalactiae, is a leading cause of invasive infections (pneumonia, septicaemia, and meningitis) in the neonate, and a serious cause of mortality or morbidity in adults with underlying diseases [19], [20]. Interestingly, this bacterium alike most if not all streptococci apparently lack the genes coding the essential enzymes for glycerophosphate WTA synthesis that are present in bacilli, enteroccci, lactobacilli, listeria, and staphylococci [21] and D-alanylation of LTAs appears to be the main mechanism of resistance to CAMPs. In order to better understand how modification of LTAs charge contributes to the resistance of GBS to CAMPs, we investigated its interaction with selected natural and de-novo designed CAMPs having different biophysical properties. For that purpose, we utilized a multidisciplinary approach combining microbiological and biophysical methods to highlight differences between various steps of the interaction of CAMPs with intact WT GBS and its isogenic dltA mutant. Our results suggest that incorporation of D-alanine into LTAs induce CAMPs resistance in GBS by modifying the rigidity and permeability of the cell wall rather than by affecting the electrostatic-driven binding of CAMPs to bacterial surface.

## Results

### The Level of Resistance Mediated by D-Alanylation of LTAs Correlates with the Length and Charge Density of CAMPs

**Figure 1:**
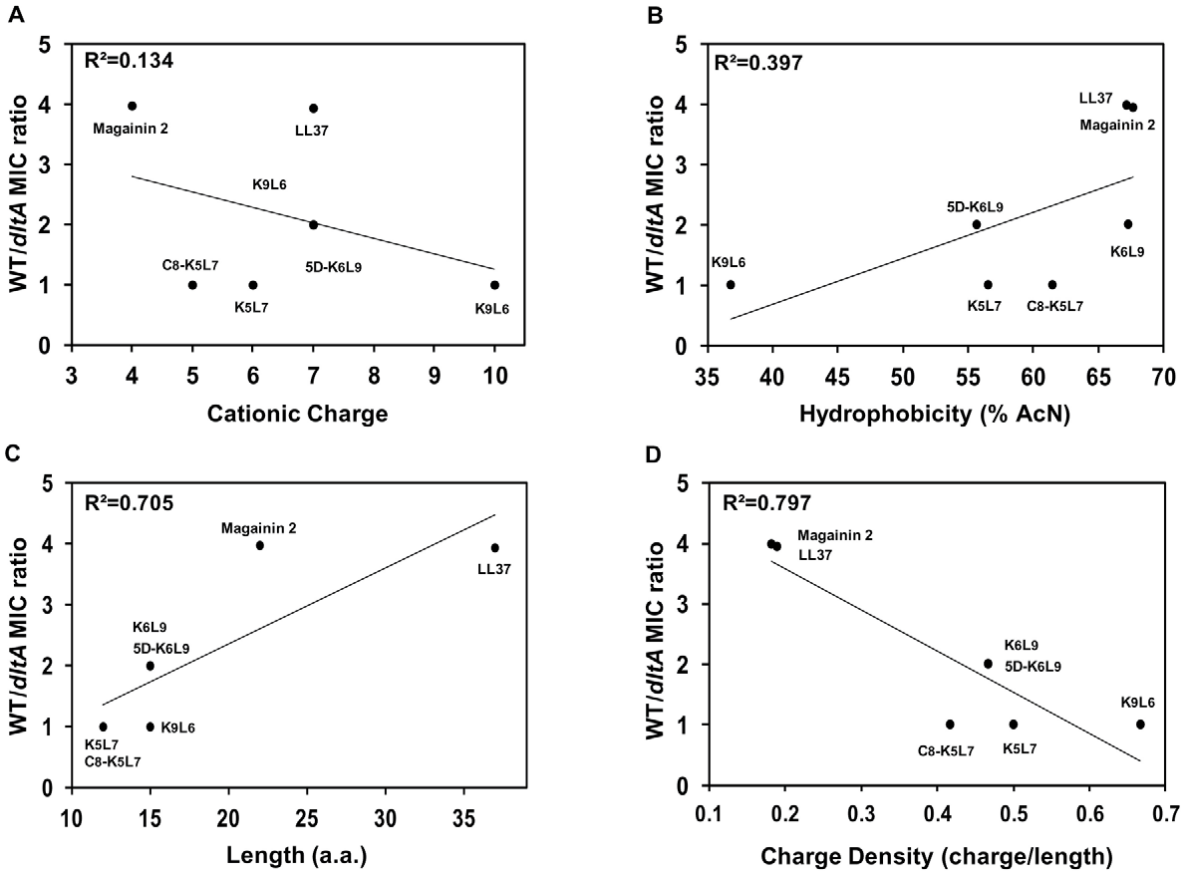
CAMPs activity against GBS strains *versus* peptide properties. Charge (A), hydrophobicity (B), length (C), and charge density (D) were plotted *versus* the ratio between the MICs against the WT strain and the isogenic *dltA* mutant. The characteristics of each peptide are shown in Table 1.

The investigated GBS strains were previously characterized revealing that 20.8% of the glycerophosphate residues of the LTAs of the WT strain NEM316 were substituted with D-alanyl esters whereas insertional inactivation of *dltA* caused complete absence of D-alanine [21]. The *dltA* mutant was highly susceptible to colistin compared to the WT strain and resistance was recovered at an intermediate level in a complemented strain where D-alanine incorporation was partially restored to 12.8%, confirming that there is a positive correlation between D-alanine content and resistance [22]. Here, we investigated whether there is a correlation between resistance and specific peptide property (net charge, hydrophobicity, charge density and length). For that purpose, a group of nine peptides having different properties was investigated. The antibacterial activity of the selected CAMPs was determined against WT GBS and its isogenic *dltA* strain. The list includes two natural linear CAMPs (magainin 2 and the human cathelicidin LL37), two natural cyclic CAMPs (polymyxin B and colistin), four well characterized short *de-novo* designed peptides, and a lipopeptide composed of lysine and leucine repeats (K_9_L_6_, K_6_L_9_, 5D-K_6_L_9_, K_5_L_7_ and C_8_-K_5_L_7_) (Table 1). Magainin 2 and LL37 are α-helical peptides with a net charge of +4 and +7, respectively [12]. K_6_L_9_ and 5D-K_6_L_9_ share the same 15 aa sequence, have a net charge of +7, but differ in their secondary structure. While K_6_L_9_ is 90% α-helical in PE/PG membrane bilayers and in lipopolysaccharide (LPS) multi-bilayers, the incorporation of D-amino acids reduced its α-helix content to 40%. This modification has been shown to improve its antibacterial activity against various gram-negative and gram-positive bacteria [23], [24]. The peptide K_9_L_6_ is highly cationic (+10) but exhibits poor antibacterial activity. Similar activity was found for K_5_L_7_, but coupling of a short fatty acid (C_8_-K_5_L_7_) strongly improved its antimicrobial activity [25]. The antimicrobial activities of all the peptides against GBS NEM316 WT strain and relevant isogenic mutants are summarized in Table 2. The data reveal that, irrespective of their net positive charge ranging from +4 to +7, all natural peptides (magainin 2, LL37, colistin and polymyxin B) are significantly more active against the *dltA* mutant (more than a 2-fold dilution) compared to the WT. In contrast, the activities of the designed peptides against the WT and *dltA* mutant strains are similar regardless of their charge (ranging from +5 to +10). We therefore try to hierarchize the biophysical properties that dictate the activity of the tested linear peptides by analyzing the minimal inhibitory concentration (MIC) ratios between the WT and *dltA* mutant (Figure 1). Interestingly, in contrast to what is expected, increased activity against the mutant does not correlate with peptides net charge. For example, LL37 is more active on the mutant compared to the WT. In contrast, the similarly charged (+7) K_6_L_9_ or 5D-K_6_L_9_ peptides display the same activity on both strains. In addition, magainin 2 is also 4-fold more active on the *dltA* mutant compared to the WT although its net charge is only +4 (Figure 1A). Morever, despite its +10 net charge, K_9_L_6_ displays weak activity on both strains. This could be due to its low hydrophobicity resulting in reduced ability to disrupt the membrane. However, there is no obvious correlation between hydrophobicity and antibacterial activity. For example, the peptides LL37, magainin 2, K_6_L_9,_ and the lipopeptide C_8_-K_5_L_7_ have similar high hydrophobicity, but only the first two are more active on the *dltA* mutant (Figure 1B). Interestingly, the MIC ratios obtained with linear cationic peptides (+4 to +10), display a significant positive correlation with the peptide length (R^2^ = 0.705) but a significant negative correlation (R^2^ = 0.797) with the charge density (Figure 1C and 1D, respectively). The data suggest that if two peptides of different sizes possess an identical cationic charge, the activity of the longer peptide is more impaired by LTA D-alanylation. Note that in comparison to the α-helical LL37 and magainin 2, the cyclic peptides polymixin B and colistin have high charge density but are 8-fold more active against the *dltA* mutant. This is probably attributed to different mechanisms of actions which involve specific binding to lipid II rather than membrane perforation [4], [26], [27].

**Table 1:** Peptide designation and properties.

Peptide designation	Sequence^a^	Net charge	Molecular mass (g/mole)	Charge density^b^	Hydrophobicity (% AcN)^c^
K_9_L_6_	LKKLKKLLKKLKKKL-NH2	+10	1849.7	0.667	36.7
5D-K_6_L_9_	LK**L**LK**K**L**LK**KLL**K**LL-NH_2_	+7	1805.4	0.467	55.6
K_6_L_9_	KLLKKLLKKLLKLL-NH_2_	+7	1805.4	0.467	67.2
K_5_L_7_	KKLLKLLLKLLK-NH_2_	+6	1451.0	0.500	56.5
C8-K_5_L_7_	CH_3_(CH_2_)_6_CO-KKLLKLLLKLLK-NH_2_	+5	1564.0	0.416	61.4
LL-37	LLGDFFRKSKEKIGKEFKRIVQRIKDFLRNLVPRTES-NH_2_	+7	4493.3	0.324	67.6
Magainin 2	GIGKFLHSAKKFGKAFVGEIMNS-NH_2_	+4	2466.9	0.227	67.1
Polymyxin B	C_50_H_100_N_16_O_17_S	+5	1301.5	0.455	53.2
Colistin	C_52_H_98_N_16_O_13_	+5	1155.5	0.455	ND

a Underlined and bold amino acids are D-enantiomers. All linear peptides are amidated in their C-terminus.

b Calculated by dividing the net charge by the total number of amino acids.

c The peptides were eluted in 40 min using a linear gradient of acetonitrile (AcN) from 30 to 70% v/v in water containing TFA (0.05% v/v) on a C4 reverse analytical column.

**Table 2:** Minimum inhibitory concentrations (MIC_90_) towards GBS strains mutated in various cell surface components.

	WT	Δ*dltA*	Δ*mprF*	Δ*lgt* Δ*lsp*	Δ*cpsD*	Δ*srtA*
K_9_L_6_	25	25	ND	ND	ND	ND
5D-K_6_L_9_	4.4	2.2	4.4	4.4	4.4	4.4
K_6_L_9_	35.5	17.7	17.7	17.7	35.5	35.5
K_5_L_7_	25	25	ND	ND	ND	ND
C_8_-K_5_L_7_	3	3	ND	ND	ND	ND
LL-37	>14.2	3.6	>14.2	>14.2	>14.2	>14.2
Magainin 2	>25.5	6.4	>25.5	>25.5	>25.5	>25.5
Polymyxin B	92.4	5.8	92.4	92.4	92.4	92.4
Colistin	>221.6	13.9	>221.6	>221.6	>221.6	>221.6

The MIC (µM) of each antimicrobial peptide is an average of triplicate measurements performed by a dilution method in 96-well polypropylene microplate. Plates were incubated overnight at 37°C and were then read at OD_600 nm_ for bacterial growth. The MICs_90_ was considered to be the peptide concentration that inhibited growth of 90% of the tested strains.

### D-alanylation of LTAs Is the Main Mechanism of Resistance to CAMPs in GBS

Since CAMPs are thought to interact with cell surface components prior to their incorporation into the membrane, we investigated whether other surface modifications beside LTAs D-alanylation could affect peptide-cell wall interaction. To address this question, peptides were tested for their activities against isogenic mutants that were unable to (i) express the transmembrane protein MprF (Gbs2090) thought to incorporate L-lysine into membrane phosphatidylglycerol, (ii) synthesize membrane-bound lipoproteins (*lgt*, *lsp*) or the capsular polysaccharide (*cpsD*), or (iii) anchor LPXTG proteins to peptidoglycan (*srtA*). Our results show no significant difference in the MICs of the WT compared to these mutant strains (Table 2), suggesting that the corresponding GBS surface components do not significantly contribute to CAMPs resistance under our experimental conditions and do not affect peptide-wall interactions. Thus, in GBS, resistance to CAMPs is mainly due to D-alanylation of LTAs.

### D-Alanylation of LTAs Protects against the Membranolytic Effect of CAMPs

**Figure 2:**
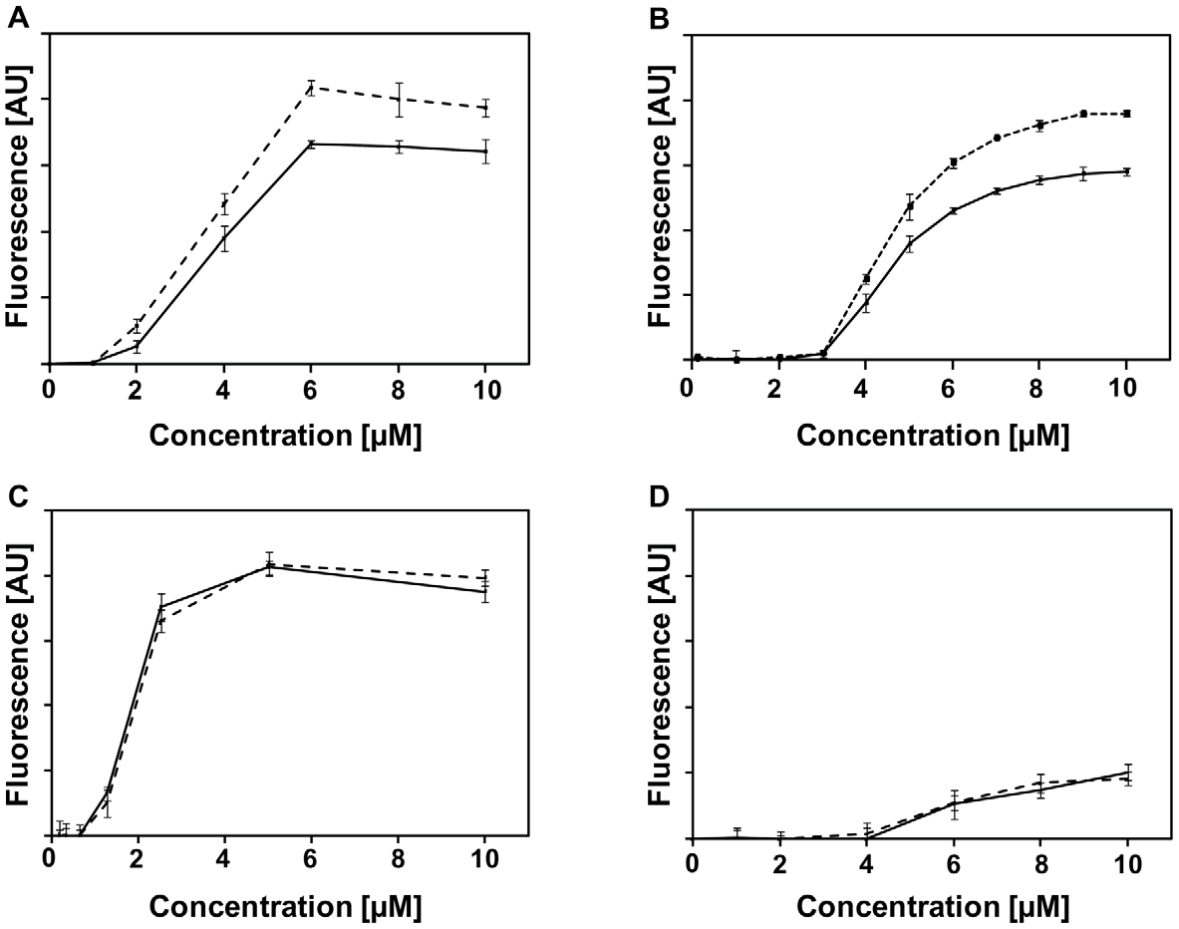
Susceptibility of GBS strains to perforation by CAMPs. Peptide-dependent influx of the vital dye SYTOX green following 30 min exposure of GBS to magainin 2 (A), LL-37 (B), 5D-K_6_L_9_ (C) and K_5_L_7_ (D). Solid lines represent the WT strain whereas dashed lines represent the *dltA* muatnt. All readings were normalized by subtracting the basal fluorescence of the dye. Data are means ± SD of triplicate measurements.

Many CAMPs exhibit their antibacterial activity through direct interaction with and subsequent perforation of the membrane. To determine whether altered susceptibility to CAMPs correlates with changes in membrane permeability, we measured the entrance of the cationic dye SYTOX green into the WT and *dltA* strains. SYTOX green cannot enter intact bacteria unless the membrane is disrupted by external compounds. Upon penetration into bacteria and binding to intracellular nucleic acids, its signal increases drastically. A dose-dependent enhancement of signal intensity was found following exposure to selected CAMPs. In agreement with the MIC assay, only magainin 2 and LL37 induced significantly stronger signal with the *dltA* mutant, compared to the parental strain (Figure 2A and 2B). Expectedly, the highly potent 5D-K_6_L_9_ was similarly active on both strains (Figure 2C) whereas the weakly potent K_5_L_7_ induced an equally low signal (Figure 2D). These results confirm that D-alanylation of LTAs protects the membrane from perturbation by CAMPs.

### The Anionicity of the Cell Surface Does Not Modulate the Accumulation of CAMPs

**Figure 3:**
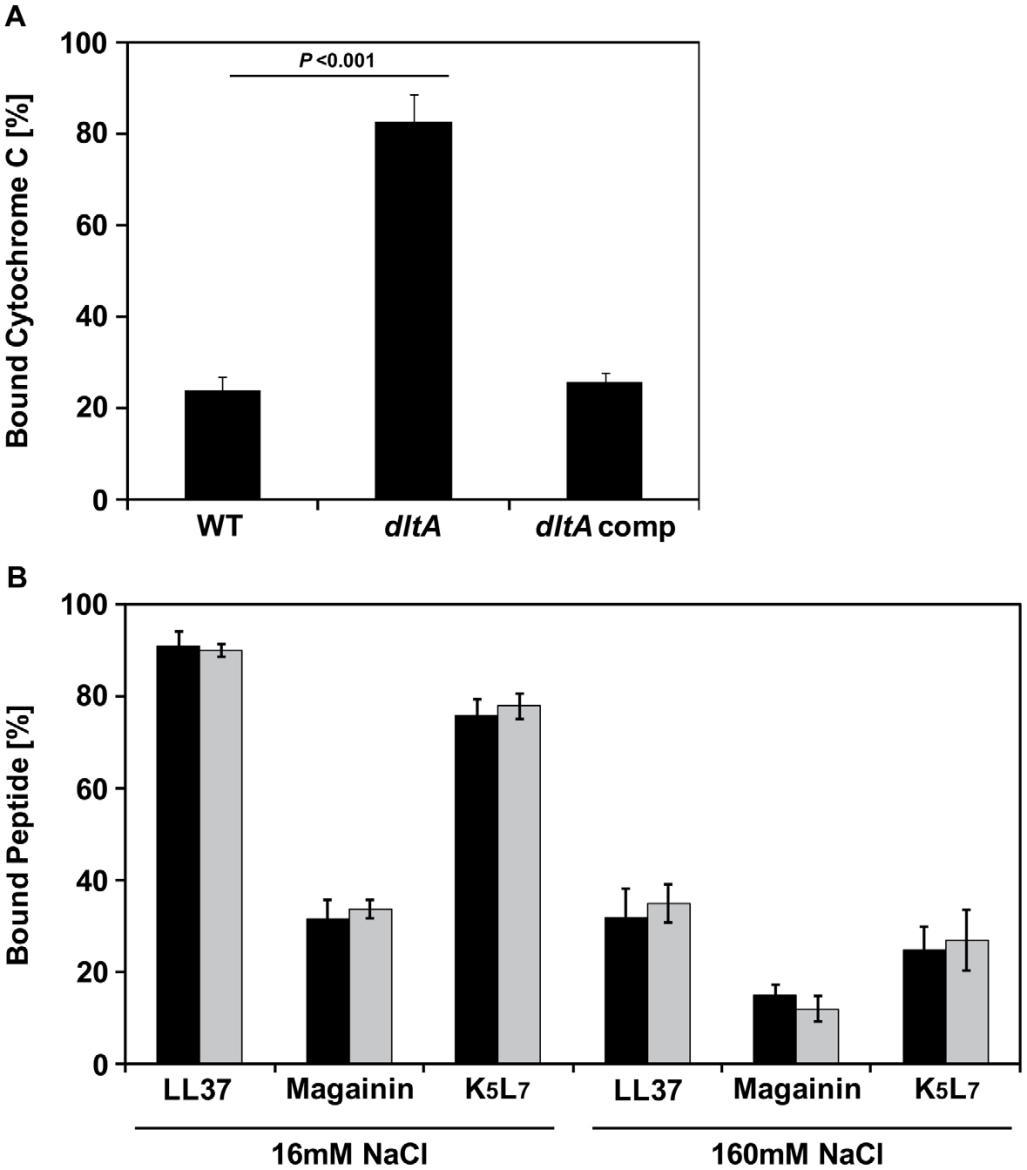
Quantitative binding assay. The binding of cytochrome C (A) and NBD-labeled CAMPs (B) to WT (black) and *dltA* (gray) mutant strains was studied. Data are means ± SD of triplicate measurements from three independent experiments and are presented as the percentage of the maximum signal (peptide with no bacteria) ± SD.

It is commonly admitted that esterification of WTAs and LTAs with D-alanyl esters reduces the surface cationic binding capacity, thereby leading to a reduced electrostatic binding of CAMPs and to an increased resistance [28]. GBS is devoid of WTAs but possess a branched rhamnose-rich carbohydrate similarly anchored to the peptidoglycan (the so-called group B antigen) [29]–[31]. This polysaccharidic sructure also displays an anionic character conferred by its high phosphate content which can mask differences in surface charge due to D-alanylation of LTAs. We therefore evaluated differences between the surface charge of GBS WT and *dltA* mutant strains by measuring their ability to bind the cationic protein cytochrome C [14]. To confirm the role of DltA in charge modification, an isogenic complemented *dltA* (*dltA* comp.) strain was also tested. The data reveal a 3-fold increased binding of cytochrome C to the *dltA* mutant compared to the WT strain and that complementation restored cytochrome C binding to the WT level (Figure 3A). A similar trend was found in *S. aureus* and Group A *Streptococcus* [14], [15]. The binding of CAMPs to the bacteria was then tested under high (160 mM NaCl) and low (16 mM NaCl) ionic strength conditions. The data reveal a direct correlation between the charge of the peptides and their binding capacity: LL37 (+6)>K_5_L_7_ (+5)>Magainin 2 (+3) in both conditions (Figure 3B). In these experiments, the peptides labeled with NBD at their N-terminus possess the net charge of the unlabeled peptides minus one, while their antimicrobial activity was unchanged (data not shown). As expected, a marked reduction in binding was observed under high ionic strength buffer due to electrostatic masking of charges. Surprisingly, despite the huge difference in surface charge between the WT and the *dltA* mutant under low ionic environment, there was no significant increase in the binding of the tested peptides to the *dltA* strain. A similar result was found also for the peptides displaying no increased activity toward the mutant strain (Figure S1).

### D-Alanylation of LTAs Modifies the Density of the Cell Wall as Revealed by Transmission Electron Microscopy (TEM)

**Figure 4:**
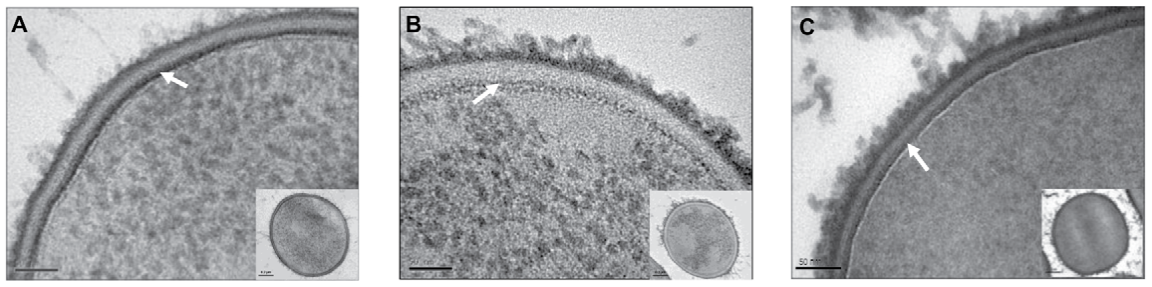
Investigation of GBS cell wall structure using transmission electron microscopy. High resolution images of the cell wall of WT (A), *dltA* mutant (B), and *dltA* complemented (C) strains, as revealed by using the freeze substitution method. The inner region of the cell wall is pointed by arrow. Bars for the main picture and for the whole cell inserted picture represent 50 nm and 200 nm, respectively.

Resistance to CAMPs could result from a thickened cell wall as demonstrated for *S. aureus* [32]. To address this possibility, we accurately measured the GBS cell wall thickness from high resolution transmission electron microscopy (TEM) images by using freeze substitution method. The morphology of the GBS cell wall is typical of that reported for other streptococci [33], being composed of two compact laminae: an intensely electron opaque inner layer that lies the cytoplasmic membrane and a less electron opaque outer layer (Figure 4). Our measurements reveal no difference between the average thickness of the WT (31.9±6.7 nm) and that of the *dltA* mutant (32.8±6.1 nm). However, the inner region of the WT cell wall was more heavily stained with metal ions (lead citrate and uranyl acetate), as compared to the *dltA* mutant strain (Figure 4). These two observations were confirmed using a *dltA* complemented strain which had similar morphology as the WT strain. The heavy staining density cannot be attributed to increase in metal binding since the WT has less anionic charge then the *dltA* strain. An alternative explanation is that higher amounts of metals are trapped in the cell wall matrix of the WT and *dltA* complemented strain [22]. Therefore, we hypothesize that D-alanylation of LTAs increases the packing density of the GBS cell wall but does not modify its thickness.

### Surface Properties of the Cell Wall Determined by Atomic Force Microscopy (AFM)

**Figure 5:**
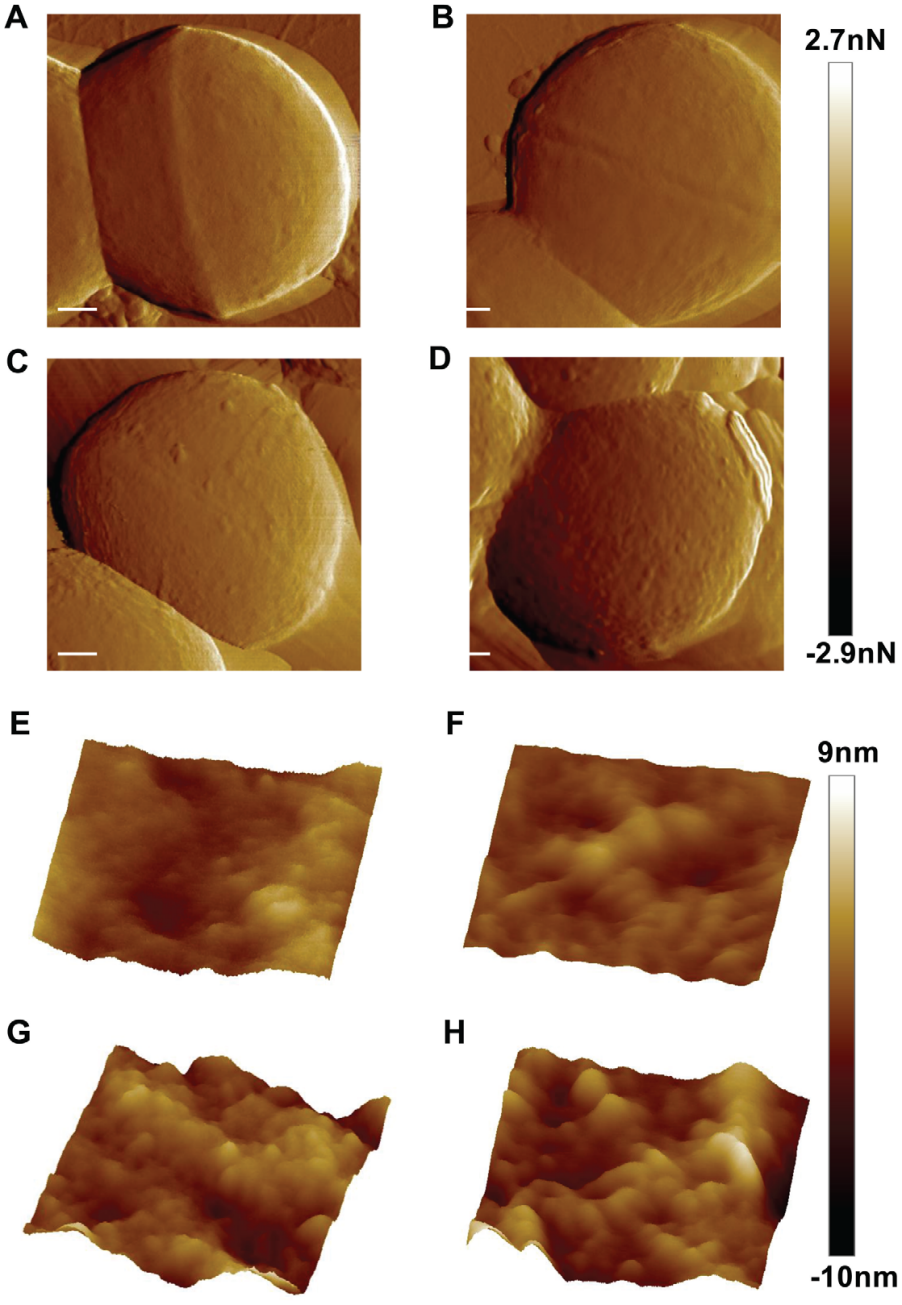
Investigation of GBS morphology using atomic force microscopy. Deflection (A–D) and topography (E–H) images of GBS surface morphology. Representative images of non-treated WT (A and E), *dltA* mutant (B and F), and LL37-treated (10 µM) WT (C and G) and *dltA* mutant (D and H).

**Figure 6:**
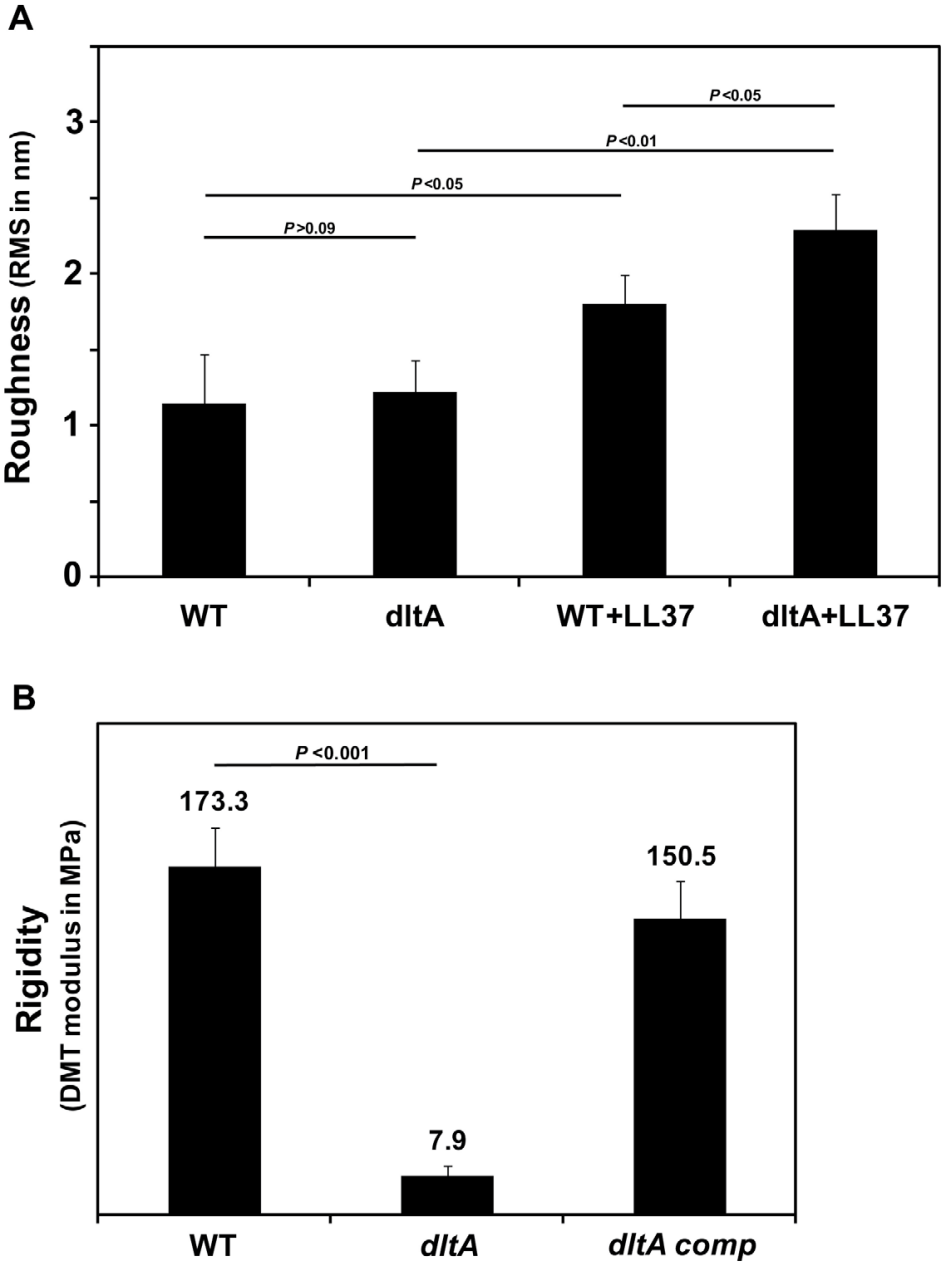
Investigation of GBS cell wall properties using atomic force microscopy. The root mean square (RMS) of surface roughness calculated from topography images (A) and the average surface rigidity calculated from DMT modulus images (B) are shown. Data are means ± SD of n≥8 cells.

We used AFM to investigate the influence of D-alanylation of LTAs on the mechanical properties of the cell wall. This method enables to measure nanoscale changes in bacterial surface with a minimal interference with cell integrity. When bacteria were grown without CAMPs, WT and its isogenic *dltA* strain did not exhibit any apparent difference in surface morphology, as imaged in an amplitude mode (Figure 5A and 5B) and in topography mode (Figure 5E and 5F). This observation is supported by calculation of the distribution of surface heights in WT and *dltA* mutant for which the root mean square (RMS) of the height distribution (1.14±0.32 nm and 1.22±0.21 nm, respectively) are similar (Figure 6A). Treatment with LL37 has significantly altered the morphology of WT and *dltA* mutant with a more pronounced effect on the mutant strain (Figure 5C and 5D in amplitude images and 5G and 5H in topography images). This results in an increase in the average RMS of the height distribution for the *dltA* mutant (2.28±0.24) compared to the WT strain (1.8±0.19) (Figure 6A). We also characterized the surface rigidity of non-treated WT, *dltA* mutant and *dltA* complemented strains by PeakForce QNM, which is based on a force-volume approach, and determination of the average value of DMT-modulus [34], [35]. The data reveal that the rigidity of cell wall of the WT strain is more than 20-fold higher than that of the *dltA* mutant (177.37±19.54 MPa and 7.87±0.66 MPa, respectively). Complementation with a functional *dltA* gene restored the rigidity to a value of 150.50±19.10 (Figure 6B). The value calculated for the GBS WT strain is in the range of those estimated for non-fixed air dried *Escherichia coli* and *S. aureus* [36] and live, agarose encapsulated *E. coli* (50–150 MPa), *Bacillus subtilis* (100–200 MPa) and *Pseudomonas aeruginosa* (100–200 MPa) [37].

### D-Alanylation of LTAs Reduces the Ability of CAMPs to Traverse the Cell Wall

**Figure 7:**
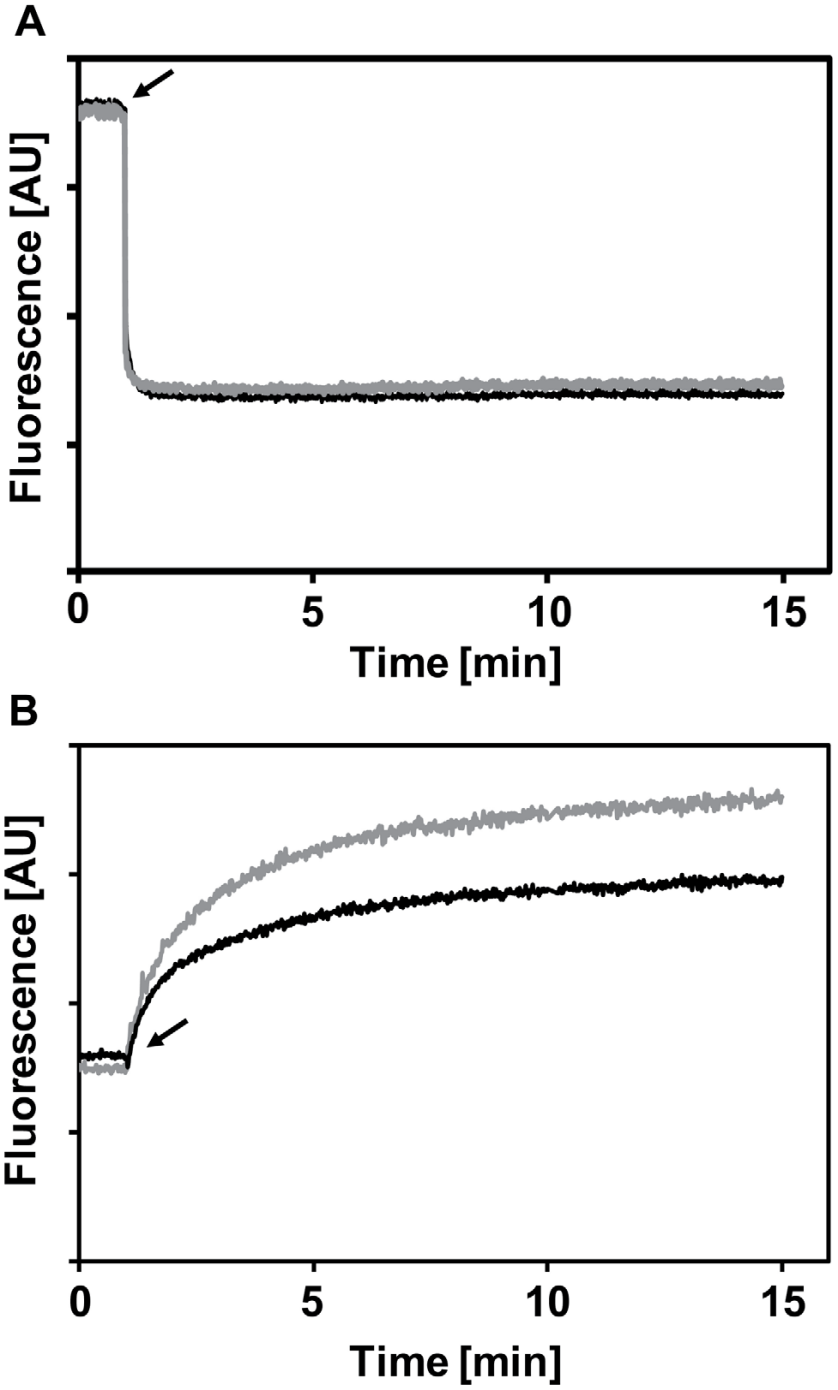
CAMPs aggregation and penetration through the cell wall. Representative curves of the change in fluorescent signal of 1 µM rhodamine-labeled (A) or NBD-labeled LL37 (B), following addition of bacteria. WT and *dltA* mutant are represented by black and gray lines, respectively. The black arrow denotes the addition of bacteria.

We further investigated whether the increased cell wall density resulting from D-alanylation of LTAs can reduce CAMPs penetration through the cell wall barrier by following the interaction of fluorescently labeled CAMPs with intact bacteria. Earlier studies demonstrated that CAMPs can aggregate upon their interaction with cell wall lipopolysaccharides [11], [23], [24], [38]. Here, we investigated if the peptides display different aggregation states when bound to the WT or the *dltA* mutant strains. This was done by monitoring changes in the signal intensity of rhodamine-labeled peptides following their interaction with the bacterial surface under under high (160 mM NaCl) and low (16 mM NaCl) ionic strength conditions. Addition of bacteria to the rhodamine-labeled peptides induced self-quenching of fluorescence for all tested peptides. However, no significant difference in aggregation was found in the WT compared to the *dltA* strain (Figure 7A and Table 3). Similar trend of aggregation was obtained when commercial LTAs isolated from *S. aureus* were used instead of intact bacteria which demonstrated that the change in aggregation state is due to the interaction of CAMPs with membrane-bound LTAs of GBS cells (Figure S2A).

**Table 3:** Aggregation and membrane interaction of CAMPs with GBS.

	Signal change [%]^a^				Difference in signal change [%]^b^	
	NaCl (16 mM)		NaCl (160 mM)		NaCl (16 mM)	NaCl (160 mM)
Peptide name	WT	*dltA*	WT	*dltA*	Δ(*dltA*-WT)	
NBD-LL37	47.0	59.1^c^	−3.4	−3.7	12.1	−0.3
NBD-Magainin 2	22.2	51.5^c^	10.3	16.1^c^	29.3	5.8
NBD-K_5_L_7_	5.3	7.1	−5.2	−4.1	1.8	−1.1
Rho-LL37	−60.8	−58.6	−26.6	−32.7^c^	−2.2	6.1
Rho-Magainin 2	−23.4	−20.2	−7.4	−6.5	−3.2	−0.9
Rho-K_5_L_7_	−41.4	−39.6	−38.9	−40.2	−1.8	−1.3

a Percentage of change in signal intensity relatively to the basal signal of the peptides.

b Difference in the signal change between *dltA* strain to the WT strain.

c Value is significantly different from that of the WT strain (P<0.05).

Data are means of triplicate measurements from two independent experiments.

We further examined the ability of the CAMPs to traverse the cell wall barrier and interact with the phospholipid membrane using NBD labeled peptides. The fluorescence emission of NBD, unlike that of rhodamine, is highly enhanced in a lipidic environment. Therefore, the signal output of NBD is strongly affected by its proximity to the membrane. We first monitored the interaction of NBD-labeled peptides with liposomes composed of cardiolipin and phosphatidylglycerol (CL∶PG), anionic lipids composing membranes of streptococci [39] but lacking all cell wall components including peptidoglycan and LTAs. Upon addition of liposomes to the NBD-labeled peptides, the signal increased rapidly due to the localization of the peptide in the hydrophobic environment of the membrane (Figure S2B). In comparison, addition of bacteria to either NBD-LL37 or NBD-magainin 2 resulted in a slower rate of signal increase (Figure 7B), suggesting that the cell wall delays the peptides from reaching the phospholipid membrane. With intact GBS cells, the signal increase due to peptide penetration at low ionic strength environment was higher for the *dltA* mutant in comparison to the WT strain, whereas only a slight differences was observed under high ionic strength for magainin 2 but not for LL37 (Table 3). In contrast, the inactive NBD-K_5_L_7_ increased its signal by only 1.8% in 16 mM NaCl and displayed a slight reduction in signal in 160 mM solution, suggesting that most of the peptide remained trapped in the cell wall and did not reach the membrane. Together, our findings suggests that electrostatic interactions between LTAs are major contributors to the ability of the bacteria to block penetration of the CAMPs.

## Discussion

The purpose of this study was to investigate the mechanism by which D-alanylation of LTAs confers resistance to CAMPs in GBS, a species chosen as a model of gram-positive bacterium devoid of WTAs. Our data will be discussed in line with the three following major findings: (i) D-alanylation of LTAs modulates the resistance towards only a subset of CAMPs whose activity is in a relation to their length and charge density; (ii) CAMPs resistance due to increased surface cationic charge is not due to decreased amounts of bound peptides to bacteria; and (iii) D-alanylation of LTAs reduces the penetration of CAMPs through the cell wall by increasing its density, most likely by altering the conformation of LTAs.

(i) Several studies have shown that inactivation of the dltABCD genes in different gram-positive species, including GBS, result in an increased susceptibility to various natural CAMPs [14], [22], [40]. Here, our data revealed a similar trend when the GBS *dltA* mutant was exposed to natural CAMPs but not to *de-novo* designed linear peptides (Table 2). Increased activity against the *dltA* mutant strain did not correlate with peptides net charge or hydrophobicity. However, reasonable correlations were found with peptides length (R^2^ = 0.705) and charge density (R^2^ = 0.797) (Figure 1). Although the main mechanism of bacterial killing of the linear peptides used in this study is membrane pertubation, deviations from the rules may be attributed to differences in the specific mode of action of each peptide. Consistently, the fact that the length and charge density of a peptide correlates with the WT/*dltA* MIC ratio, whereas its net charge does not influence its bactericidal activity, suggests the existence of at least one alternative mechanism of intrinsic resistance besides electrostatic repulsion.

The data on the efflux of SYTOX green into the non-growing cells due to treatment with CAMPs (Figure 2) confirmed that incorporation of D-alanyl resisdues into LTAs protect the membrane integrity by reducing the capability of the CAMPs to disrupt it. This observation was not due to peptide interaction with cell wall components such as the capsular polysaccharide and surface proteins. In addition, we provide evidence that under our conditions the transmembrane MprF protein thought to aminoacylate the membrane phospholipids did not significantly contribute to resistance towards the CAMPs used in this study (Table 2).

(**ii**) The activities of cationic peptides, such as nisin and gallidermin, against GBS and *S. aureus* decrease as the bacterial electropositive surface charge increases through incorporation of D-alanyl residues in LTAs [14], [22]. Note that these antibiotics target the cell wall through electrostatic interactions and dock specifically on the cell wall precursor lipid II to inhibit peptidoglycan synthesis [4], [26], [27]. Therefore, the activity of these molecules is expected to be highly affected by surface charge modification. A similar mechanism of electrostatic repulsion was demonstrated for the activity of the staphylococcal *mpr*F gene product which attenuates membrane charge [41]. However, a recent study by Kilelee et al. showed that physiological concentrations of lysyl-PG enhances the resistance of model membranes to the CAMP 6W-RP-1 due to a decrease in lipid clustering rather than by a mechanism of electrostatic repulsion [42]. Under our experimental settings, the GBS *mprF* mutant was found as resistant as the parental WT strain to all CAMPs tested in this study and this result is currently under investigation. Our results reveal a direct correlation between the net charge of the peptides and their binding capacity to either WT or *dltA* mutant strains but no significant difference was observed in the binding of a given peptide to either strains (Figure 3 and Figure S1). On the other hand, the cytochrome C protein binds in higher amounts to the *dltA* mutant compared to the WT strain which indicate that there is a significant difference between their overall surface charge. Therefore, the binding of the tested CAMPs, unlike that of cytochrome C, seems less affected by changes in GBS cell wall charge. These results suggest that charge repulsion is not the major mechanism by which D-alanylation of LTAs promotes CAMPs resistance, as shown in *S. aureus* for the human group IIA phospholipase A2 [43].

(**iii**) The secondary structure of LTAs highly depend on the presence of environmental cations. Under low ionic-strength conditions, the anionic LTA chains repel each other and therefore exist in an extended conformation as it was also proposed for LPS molecules of gram-negative bacteria [44]. In high ionic-strength solutions, it has been shown that the electrostatic screening of the poly(Gro-P) moiety of LTA by sodium ions induced random-coil conformation which in turn increased the packing density of the matrix [45]. In this model, incorporation of D-alanyl residues should mask the anionic phosphates of the LTAs to reduce repulsion between neighboring molecules [46], [47]. We postulated that this structural modification could affect the mechanical resistance of the cell wall to CAMPs. Indeed, TEM analysis showed that D-alanylation of LTAs increase the density of the cell wall (Figure 4). Moreover, AFM analysis revealed that the surface rigidity of WT GBS strain is more than 20-fold higher than that of the *dltA* mutant and that gene complementation restores rigidity to values similar to the WT strain (Figure 6B). As a likely consequence, the WT strain displays reduced alterations of its surface topography following treatment with LL37 (Figure 5) and attenuated penetration of fluorescently labeled CAMPs through the cell wall barrier (Figure 7 and Table 3). Our results show that high NaCl concentration reduces the penetration of CAMPs through the cell wall of the *dltA* strain to restore the WT behaviour (Table 3). Therefore, electrostatic screening of cell wall charge can be achieved either through D-alanylation of LTAs or by electrostatic interaction with metal cations. This delicate interplay between environmental cations and incorporation of D-alanyl residues, which could be regulated at the transcriptional level [20], [42], allow the bacteria to change the mechanical properties of its cell wall to resist CAMPs attack.

In conclusion, our work constitue a detailed investigation of the mechanism by which D-alanylation of LTAs can mediate resistance to CAMPs in GBS. Based on our observations, we suggest that D-alanylation of LTAs increase the packing of the cell wall of gram-positive bacteria to reduce the effective peptide concentration over the membrane. Our findings uncover a novel protective role of the cell wall against CAMPs and therefore constitute an advance in our understanding of bacterial defense mechanisms against these molecules.

## Materials and Methods

### Materials

Rink amide MBHA resin and 9-fluorenylmethoxycarbonyl (Fmoc) amino acids were purchased from Calibochem-Novabiochem AG (Switzerland). Other reagents used for peptide synthesis include N, N-diisopropylethylamine (DIEA, Sigma- Aldrich), dimethylformamide, dicheloromethane, and piperidine (Biolab, Israel). 4-chloro-7-nitrobenz-2-oxa-1, 3-diazole fluoride (NBD-F), rhodamine-N-hydroxysuccinimide (Rho-N) and SYTOX green were purchased from Molecular Probes (Junction City, OR, USA). LTAs from *Staphylococcus aureus* (L2515) and Cytochrome C were purchased from Sigma-Aldrich (Rehovot, Israel). Cardiolipin and phosphatidylglycerol were purchased from Avanti Polar Lipids (Alabaster, Alabama).

### Peptide Synthesis and Fluorescent Labeling

The procedure conducted as described previously [23]. Briefly, peptides were synthesized using the Fmoc solid phase method on Rink amide resin (0.68 meq/mg). For fluorescent labeling of the N-terminus of the peptides, resin-bound peptides were treated with NBD or rhodamine dissolved in dimethyl formamide (DMF), washed thoroughly with DMF and then with methylene chloride, dried and then cleaved. The peptides were purified (greater than 98% homogeneity) by reverse phase high performance liquid chromatography (RP-HPLC) on a C4 column using a linear gradient of 30–70% acetonitrile in 0.1% trifluoroacetic acid (TFA) for 40 minutes. The peptides were subjected to amino acid and mass spectrometry analysis to confirm their composition.

### Bacterial Strains

The bacterial strains used in this study and their main characteristics are listed in Table S1.

### Antibacterial Activity

The MICs of each antimicrobial peptide were tested in Todd-Hewitt broth (THB) buffered with 100 mM HEPES in 96-well polypropylene microplates (Costar, Cambridge, MA) by a dilution method. Bacteria (2×10^6^ CFU) were added in triplicates to wells containing increasing concentrations of the antimicrobial peptides. Plates were incubated at 37°C with shaking overnight and then read (OD_600 nm_) using microplate reader (Labsystems Multiskanat) for bacterial growth. The MICs_90_ was considered to be the peptide concentration that inhibited 90% growth.

### SYTOX Green Uptake Assay

Bacteria were washed twice, resuspended in sodium phosphate buffer (SPB, 20 mM, pH 7.4), and incubated with 1 µM SYTOX green for 20 min in the dark with agitation [48]. Bacteria were added to a 96 well black plate (Nunc, Denmark) containing increasing concentrations of CAMPs and the increase in fluorescence, due to penetration and binding of the dye to intracellular DNA, was immediately monitored (excitation of 485 nm and emission of 520 nm). Results are mean values ±SD of three independent experiments, performed in triplicates.

### Surface Charge and CAMPs Binding

Bacteria were harvested and washed twice with PBS (16 mM, pH 7.2). Cells from 200 µl aliquots (OD_600 nm_ adjusted to 4) were incubated (shaking in dark) with 0.5 mg/ml Cytochrome C. For peptide binding, 200 µl bacterial aliquots (OD_600 nm_ adjusted to 0.75) were incubated with NBD labeled peptides (1 µM). Samples were centrifuged (14,000 g, 3 min) and 100 µl of the supernatant were collected and assayed photometrically (Cytochrome C, 530 nm) or fluorescently (NBD excitation at 467 nm and emission at 530 nm). In order to dissolve any peptide aggregates that may quench the fluorescent signal, 50 µl of 6 M guanidine hydrochloride were added to the NBD-peptide wells. Data is presented as a percentage of the maximum signal (peptide only) ± SD of three independent experiments, carried out in triplicate.

### CAMP -Membrane Interactions Using Fluorescence Spectroscopy

Real time tracking of the changes in the fluorescent signal of rhodamine or NBD labeled peptides was performed using an SLM-Aminco Bowman series 2-luminescence spectrophotometer (SLM-Aminco, Rochester, NY, USA) at room temperature. Typical spectral bandwidths were 5 nm for excitation and 5 nm for emission. The ability of the peptides to traverse the cell wall and interact with the membrane was evaluated using 1 µM peptide, a concentration that did not disrupt membrane integrity in the SYTOX green experiments. The peptides were placed in a 5×5-mm quartz cuvette with constant magnetic stirring. Following signal stabilization, bacterial suspension (10 µl, OD_600 nm_ adjusted to 4) or liposomes suspension (100 µM lipid final concentration) composed of cardiolipid/PG (1∶1 M/M) [49] was added to the cuvette and the change in signal was monitored over time.

### Atomic Force Microscopy

AFM imaging was as described previously [50]. Prior to the measurements, bacterial culture was washed, resuspended in PBS and adjusted to OD_600 nm_ of 0.5. Bacteria were immobilized on freshly cleaved MICA coated with poly-L-lysine (0.01 mg/ml), washed to remove unattached bacteria, and gently fixated with 1.5% glutaraldehyde for 10 min. Samples were washed again with DDW to remove glutaraldehyde traces and left to dry overnight at room temperature. Images of bacteria were acquired with MultiMode AFM (Bruker, Santa Barbara, CA) equipped with the Nanoscope V controller and a small scanner. Images were recorded in air, at room temperature (22–24°C), in PeakForce QNM (quantitative nanomechanical mapping) mode using silicon nitride WSlevers (ORC8-PS-W, Olympus) with a nominal spring constant of 0.76 N/m. PeakForce QNM AFM imaging mode yields quantitative nanomechanical mapping of material properties, including DMT modulus and adhesion. In the same time sample topography is imaged with high resolution (1024 pixels) and minimized sample distortion due to the fine adjustment of the force applied to the sample surface. The applied force was adjusted around 1 nanonewton and the scan rate was set to 1 Hz. Imaging was carried out at different scales to verify the consistency and robustness of the evaluated structures. Numerical data presented is the mean value (±SD) of the root-mean-square (RMS) of surface roughness or DMT modulus of four 150×150 nm samples, taken from a 700×700 nm field (n≥8 cells for each treatment).

### Transmission Electron Microscopy

Cells were centrifuged and the pellet was loaded on aluminum discs with depth of 100 µm (Engineering Office M. Wohlwend GmbH, Switzerland) and covered with a flat disc. The sandwiched sample was frozen in a HPM010 high-pressure freezing machine (Bal-Tec, Liechtenstein). Cells were subsequently freeze-substituted in a AFS2 freeze substitution device (Leica Microsystems, Austria) in anhydrous acetone containing 2% glutaraldehyde and 0.2% tannic acid osmium tetroxide for 3 days at −90°c and then warmed up to −30°c over 24 hours. Samples were washed three times with acetone, incubated for one hour at room temperature with 2% osmium tetroxide, washed three times with acetone and infiltrated for 5–7 days at room temperature in a series of increasing concentration of Epon in acetone. After polymerization at 60°c, 60–80 nm sections were stained with uranyl acetate and lead citrate and examined in a Tecnai T12 electron microscope (FEI, Holland) operating at 120 kV, utilizing a 2k by 2k ES500W Erlangshen CCD camera (Gatan, UK).

### Statistical Analysis

Results were analyzed using a single factor ANOVA test. Values of p<0.05 were considered statistically significant.

## Supporting Information

Figure S1 **Quantitative binding of all tested CAMPs.** The binding of NBD-labeled CAMPs to WT (black) and *dltA* (gray) mutant strains was studied. Data are means ± SD of triplicate measurements from three independent experiments and are presented as the percentage of the maximum signal (peptide with no bacteria) ± SD.
(EPS)

Figure S2 **CAMPs aggregation and membrane interaction.** Investigation of rhodamine-labeled peptide aggregation upon interaction with *S. aureus* LTAs (A) and NBD-labeled peptide with cardiolipin: PG liposomes (B). The black arrow denotes the addition of LTA (A) or liposomes (B).
(EPS)
