# Correction: D-Alanylation of Lipoteichoic Acids Confers Resistance to Cationic Peptides in Group B *Streptococcus* by Increasing the Cell Wall Density

**DOI:** 10.1371/annotation/05894f00-6d95-4b7a-aff1-2e008d2a864f

**Published:** 2012-11-05

**Authors:** Ron Saar-Dover, Arkadi Bitler, Ravit Nezer, Liraz Shmuel-Galia, Arnaud Firon, Eyal Shimoni, Patrick Trieu-Cuot, Yechiel Shai

Table S1, "Bacterial strains used in this study," does not appear. Please view Table S1 here: 
