# Supplementary material for: Correction: D-Alanylation of Lipoteichoic Acids Confers Resistance to Cationic Peptides in Group B Streptococcus by Increasing the Cell Wall Density
Source: PLoS Pathog. 2012 Nov 5;8(11):10.1371/annotation/05894f00-6d95-4b7a-aff1-2e008d2a864f. doi: 10.1371/annotation/05894f00-6d95-4b7a-aff1-2e008d2a864f (PMC3552663; doi:10.1371/annotation/05894f00-6d95-4b7a-aff1-2e008d2a864f)
Supplement: Supplementary file 1 [file ppat.05894f00-6d95-4b7a-aff1-2e008d2a864f.s001.docx]

**Table S1**. Bacterial strains used in this study.

| Source | Relevant genotype and characteristics^a^ | Strains |
| --- | --- | --- |
|  |  | *S. agalactiae* |
| [[51](#_ENREF_51)] | WT strain, serotype III, ST23 | NEM316 |
| [[19](#_ENREF_19)] | NEM316 *dltAΩaphA-3*; Km | *dltA* |
| This study | NEM316 with an in-frame deletion of *mprF* (gbs2090) | *mprF*^b^ |
| [[52](#_ENREF_52)] | NEM316 *lgtΩaphA-3* *lspΩaad6*; Km, Sm | *lgt-lsp* |
| [[19](#_ENREF_19)] | NEM316 *cpsDΩaphA-3*; Km | *cpsD* |
| [[51](#_ENREF_51)] | NEM316 *srtAΩaphA-3*; Km | *srtA* |

*^a^aphA-3*, 3'-aminoglycoside phosphotransferase type III; *aad6*, 6-adenylyltransferase; Km and Sm, enzymatic resistance to kanamycin and streptomycin, respectively; Str, streptomycin-resistant mutant.

*^b^*Deletion of *S. agalactiae mprF* gene was carried out as described [[53](#_ENREF_53)] by using splicing-by-overlap-extension PCR using primers O5 (TGATAGAATTCTATTGGTAGGCCATAGCG) plus O6 (GGCACGCCCGGGTGCTGCCGCTAGTTGCTCAACAGAAATAGTTCG) and O7 (GCGGCAGCACCCGGGCGTGCCCGTTCGTCCTGGTTAATTTGTGCT) plus O8 (TTTAAGGATCCAGAGGATGGGAGTATTGGT) and confirmed by PCR and sequence analysis. In this mutant, 92% of *mprF* was in-frame deleted.
